# Follicular helper-like γδ T cells promote plasma cell differentiation in Behçet’s disease

**DOI:** 10.3389/fimmu.2026.1763174

**Published:** 2026-02-09

**Authors:** Sahar Shaaban Mohammed, Abdullah Khalifah S. Alkhalifah, Rahilah Mirza, Sarah-Pristine Omoefe Okinedo, Rani Aishwarya Inampudi, Azimoon Bibi, Amal Senusi, Claire Pardieu, Neil E. McCarthy, Farida Fortune, Fabian Flores-Borja

**Affiliations:** 1 Centre for Oral Immunobiology and Regenerative Medicine, Institute of Dentistry, Faculty of Medicine and Dentistry, Queen Mary University of London, London, United Kingdom; 2 Medical Microbiology and Immunology Department, Faculty of Medicine, Minia University, Minia, Egypt; 3 Department of Oral and Maxillofacial Diagnostic Sciences, College of Dentistry, Qassim University, Buraydah, Saudi Arabia; 4 Behçet’s Centre of Excellence, Royal London Hospital, Barts Health NHS Trust, London, United Kingdom; 5 Centre for Immunobiology, The Blizard Institute, Faculty of Medicine and Dentistry, Queen Mary University of London, London, United Kingdom

**Keywords:** autoantibody production, Behçet’s disease, heat shock protein 60, plasma cells, γδT-cells and B cell interaction, ICOS, CXCR5, PD-1

## Abstract

**Objectives:**

Behçet’s disease (BD) is a systemic vasculitis characterized by recurrent oral and genital ulcers. The disease can manifest diverse phenotypes -such as mucocutaneous, ocular BD- with an uncertain role for autoantibodies in disease pathogenesis. Altered γδT-cell and B-cell phenotypes have been widely reported in BD, but it remains unknown whether these lineages can interact to promote autoantibody production.

**Methods:**

This study included 75 patients with a BD diagnosis, alongside 41 healthy control (HC) volunteers. We performed *ex vivo* flow-cytometric profiling of blood γδT and B cells, established a cell culture system to investigate plasma cell generation *in vitro*, and quantified anti-HSP60 autoantibody levels in BD and HC participants’ serum and cell culture supernatants.

**Results:**

BD patients with active disease displayed a significant increase in the frequency of cells CXCR5^+^PD-1^+^ Vδ2 T cells resembling a follicular helper-like functional state. Upon stimulation, Vδ2 T cells from BD patients showed increased expression of ICOS and CXCR5, induced significant B cell proliferation, and promoted differentiation of plasma cells *in vitro*. Cultures of cells from BD patients contained increased levels of multiple cytokines that can support plasma cell differentiation (IL-4, IL-10, IL-17, CXCL13, TNF-α, IFN-γ). Anti-HSP60 autoantibodies were significantly enriched in blood serum from BD patients with active disease as well as the supernatants of patient-derived cell cultures compared to the healthy volunteer cell cultures.

**Conclusion:**

Our findings suggest that γδT cells may enhance B-cell differentiation into antibody-producing plasma cells in BD patients with mucocutaneous and ocular clinical phenotypes.

## 1 Introduction

Behçet’s disease (BD) is a systemic vasculitis of obscure aetiology characterised by recurrent oral and genital ulcers, as well as distinct ocular, vascular, and neurological manifestations, which can lead to chronic morbidity and 5% mortality rate if untreated (1). Onset of BD is thought to be triggered by infection and/or environmental factors that disturb immune homeostasis in genetically susceptible individuals, leading to systemic inflammation and tissue damage (1, 2).

Both innate and adaptive immunity are involved in BD pathogenesis (1) with associated phenotypic and functional abnormalities widely reported in both T and B-cell compartments (3). More recently, ‘unconventional’ γδT-cells have been implicated as key effectors in BD (2). Human γδT-cells are classified into a Vδ1 subset that is enriched at epithelial barriers, and a Vδ2 subset that predominates in blood (4), where they bridge innate and adaptive immune systems (5). In particular, Vδ2T-cells are uniquely responsive to non-peptide ‘phosphoantigens’ such as (E)-4-hydroxy-3-methyl-but-2-enyl pyrophosphate (HMB-PP) and isopentenyl pyrophosphate (IPP) derived from pathogenic bacteria and stressed/transformed human cells, respectively (6). Depending on their mode of activation, Vδ2T-cells direct conventional lymphocytes to adopt distinct functional profiles that can modify immunity at mucosal barrier sites (7), which could play an important role in shaping BD pathophysiology (1).

Previous studies have reported that γδT-cells can influence patterns of antibody expression in both mice and humans (8–11), but the mechanisms by which this population modifies B-cell function are not fully studied (5, 8, 9). Abnormal frequencies of B-cells and increased antibody production have also been reported in BD (2, 12). Proposed interactions between these lineages resemble classical crosstalk of T follicular helper (Tfh) cells with B-cells, in which direct provision of co-stimulatory signals and cytokines (e.g. IL-4, IL-10, IL-21) induce B-cell differentiation and antibody production (5). Indeed, γδT-cells express key mediators of interaction with B-cells, including inducible costimulatory molecule (ICOS), chemokine receptor 5 (CXCR5), programmed cell death-1 protein (PD-1), and CD40L (13, 14).

To date, only a limited number of autoantigens have been implicated in BD pathogenesis, including the disease-specific protein CTDP-1 (15), as well as autoantibodies against a range of targets such as heat shock protein 60 (HSP60) (16–19). HSP60 is an intracellular chaperonin protein can be over-expressed and redistributed to the cell surface as a ‘danger signal’ under conditions of stress, which has also been described in active BD lesions (20, 21). Notably, both B-cells and Vδ2T-cells can recognise mycobacterial and streptococcal-derived HSP65 (3, 22, 23), which is a close molecular mimic of human HSP60 (24, 25). It is therefore feasible that recognition of bacterial HSP65 could induce host responses against human HSP60 and thereby promote autoantibody generation in BD (26).

While γδT-cells appear to play a key role in BD pathogenesis, their potential contribution to autoantibody production in affected patients remains poorly defined. This study aimed to investigate potential B and Vδ2T-cell interactions and resulting effect of autoantibody production. We employed a combination of *ex vivo* immunophenotyping and *in vitro* cell culture methods together with quantification of anti-HSP60 autoantibody levels in patient serum samples and cell culture supernatants.

## 2 Methods

### 2.1 BD patient and healthy control volunteers

Our cohort included n=75 BD patients with mucocutaneous or ocular clinical manifestations attending the Behçet’s Centre of Excellence at the Royal London NHS Trust. BD diagnosis met International Criteria 2014 for BD (27) (**Table 1**). Age and gender-matched adult healthy controls (HC, n=41) were recruited alongside, in good general health and had no symptoms of infection for at least 3 weeks prior to blood collection. The study was conducted in compliance with the Helsinki Declaration and under Ethical Approval P/03/122 granted by The Queen Mary Research Ethics Committee and City Research Ethics Committee. All patients were stratified according to their disease activity state (active/inactive) and clinical phenotypes (mucocutaneous/ocular), based on their BD current activity form (BDCAF) score and clinical evaluation at the time of their visit to the clinic and blood sample collection. All participating patients and healthy volunteers gave their written informed consent prior to taking part in the study.

**Table 1:** BD patient demographic and clinical data.

Characteristics	BD patients		
	Total patients	Mucocutaneous	Ocular
Cohort number: n (%)	75 (100)	36 (48)	39 (52)
Age (Mean ± SD years)	37.9 ± 9.3	41.2 ± 9.9	34.66 ± 7.4
	n (%)		
Sex			
Male patients	36 (48)	9 (25)	27 (75)
Female patients	39 (52)	27 (69.2)	12 (30.8)
Geographical region			
White British	46 (61.3)	25 (54.3)	21 (45.7)
Turkish	8 (10.7)	2 (25)	6 (75)
Other Southern European	5 (6.7)	–	5 (100)
Asian	3 (4)	1 (33.3)	2 (66.7)
African	1 (1.3)	1 (100)	–
Undisclosed	12 (16)	7 (58.3)	5 (41.7)
Disease activity			
Active BD	28 (37.3)	14 (50)	14 (50)
Inactive BD	47 (62.7)	22 (48.9)	25 (51.1)
HLA-B51 positive*	16 (38.1)	5 (31.25)	11 (68.75)
Current Medications			
Biological options	22 (30.6)	9 (40.9)	13 (59.1)
•Infliximab	20 (90.9)	8 (40)	12 (60)
•Vedolizumab	2 (9.1)	1 (50)	1 (50)
Steroids	27 (36)	8 (29.6)	19 (70.4)
Azathioprine	38 (50.7)	15 (39.5)	23 (60.5)
Colchicine	43 (57.3)	21 (48.8)	22 (51.2)
C-reactive protein > 10 mg/L	10 (13.3)	4 (40)	6 (60)
Erythrocyte sedimentation rate > 15 mm/hour	24 (32)	12 (50)	12 (50)

*HLA-B51 data available for 42 patients.

### 2.2 Peripheral blood mononuclear cells isolation and serum collection

Peripheral blood samples (25-30mL) were collected from BD patients and HC into EDTA-vacutainers (Becton Dickinson). Peripheral blood mononuclear cells (PBMCs) were isolated by density gradient centrifugation over Ficoll-Paque™Plus (GE Healthcare) and suspended in foetal bovine serum (FBS, Thermo Fisher Scientific) containing 10% dimethyl sulfoxide (Sigma), 100 IU/mL penicillin, and 100 mg/mL streptomycin (Gibco) for cryopreservation and storage in liquid nitrogen until subsequent use. For serum samples, 5mL of peripheral blood was collected in ‘clot activator’ vacutainers. Tubes were centrifuged for 5 min at 1500xg before serum was collected and stored at -80 °C until used.

### 2.3 Flow cytometry

Phenotyping of B and T cell subpopulations was carried out using PBMCs directly *ex vivo* or after *in vitro* cell culture. For membrane markers, cells were stained with a Live/Dead discriminant dye (BioLegend), followed by blocking with Fc receptor binding inhibitor Human TruStain FcX™, (BioLegend). The cells were then incubated with different cocktails of fluorochrome-conjugated antibodies (**Supplementary Tables 1**-**3**), washed in MACS buffer, and fixed with 2% paraformaldehyde (PFA). For analysis of intracellular cytokines or intranuclear markers, after membrane staining, cells were fixed/permeabilised with Cyto-Fast™ Fix/Perm Buffer Set (BioLegend) or True-Nuclear™ Transcription factor buffer set (BioLegend) respectively and incubated with specific antibodies as listed in **Supplementary Table 4**-**6**. Data were acquired with FACSDiva or SpectroFlow^®^ software on LSRII or Aurora (Cytek) (Beckton Dickinson) cytometers respectively. Live single cells were gated on FSC-A versus SSC-A and SSC-A versus Live/Dead dye FACS plots. Fluorescence-minus-one control samples were used for adjustments of gates. Data were analysed using FlowJo v10.10 (TreeStar).

### 2.4 Cell cultures

Frozen PBMC from BD patients and HC were thawed and treated with 100mg/mL DNase I (Merck) for 10 minutes, washed in RPMI medium, and adjusted to a concentration of 1x10^6^cell/mL in serum-free RPMI medium. For proliferation analyses, PBMC were stained with 3µM CMFDA cell tracker™ green dye. Then cells were then washed, resuspended in complete RPMI medium [containing 20% FBS (Thermo Fisher Scientific), 100IU/mL penicillin, 100mg/mL streptomycin (Gibco); 5mM L-glutamine (Gibco) and 0.1mM non-essential amino acids (Gibco)] and plated in 96-well round bottom plates at a density of 2x10^5^cells/well. For *ex vivo* intracellular cytokine evaluation: cells received 50ng/mL of phorbol 12-myristate 13-acetate (PMA) (Adipogen), and 250ng/mL of ionomycin (Iono, eBioscience), in addition to of 2µM BD Golgi Stop (Monensin) (BioLegend), and 1µL/million cells of BD Golgi Plug (Brefeldin) (BD Bioscience), and incubated for 4 hours. Vδ2T-cells were specifically stimulated within the total PBMCs (28–30), using 100ng/mL (E)-4-Hydroxy-3methyl-but-2-enyl pyrophosphate (HMB-PP) (Sigma Aldrich) and 20ng/mL IL-15 (Peprotech). Non-stimulated controls were included for each sample for comparative analysis. A positive control culture for plasma cell induction was set-up using PBMC stimulated with 5µg/mL anti-human IgG/A/M (Abcam), 2.5µg/mL CpG (Alpha Diagnostics Int), 1µg/mL soluble CD40L (Enzo Life Sciences), and 50ng/mL IL-21 (Peprotech) (31). For intracellular cytokine analysis, cells were harvested at 6, 12, and 24 hours. Cultures were maintained for 5 days at 37 °C in a humidified atmosphere with 5%CO2 and supplemented every day with 50µM β-mercaptoethanol (Sigma). Cells were collected on day 5 for staining with the γδT and B cell antibody panels (**Supplementary Tables 3**, **4**). For analysis of autoantibody levels in supernatant, cultures were maintained for 10 days (32). On day 4, cultures (both stimulated and non-stimulated) were supplemented with human recombinant (hr) IL-2 (20U/mL), IL-6 (50ng/mL), IL-10 (50ng/mL), IL-15 (10 ng/mL) (Peprotech) and mouse anti–CD40L IgG (1µg/mL; clone TRAP1, Biolegend) to sustain B cell proliferation and differentiation. On day 7, cultures were supplemented with hr IL-6 (50ng/mL), IL-15 (10ng/mL) (Peprotech), hr hepatocyte growth factor (20ng/mL) (Cell Sciences, Canton, MA), and hyaluronic acid (100µg/mL, Sigma) to promote plasma cell differentiation and viability (32). After 10 days, supernatants were collected and frozen at -80 °C for subsequent analysis. Cell viability of cultured cells was assessed by flow cytometry with Zombie Ultra-Violet Live Dead viability dye.

### 2.5 Anti-HSP60 autoantibody ELISA

Frozen serum samples from HC and BD patients were thawed, diluted 1:250 in the sample diluent buffer while cell culture supernatants were used undiluted. All samples were tested in duplicate to determine levels of anti-HSP60 IgG/A/M antibodies using commercial ELISA kits (Enzo Life Sciences) following manufacturer’s instructions. Plates were read using a Clariostar microplate reader (BMG Labtech) and absorbance recorded at 450nm. Antibody quantitation was performed by interpolation from a standard curve.

### 2.6 Multiplex cytokine arrays

The supernatants of cultured cells (for 1 day and 5 days) culture supernatants were used to evaluate cytokines concentration using the Luminex R&D Systems Discovery Assay (Biotechne). This assay was a custom-made, premixed, multi-analyte ELISA incorporating reagents for quantitative detection of IL-4, IL-10, IL-17, IL-21, CXCL13, IFN-γ, and TNF-α. Assay samples were prepared and read in MagPix Luminex instrument following the manufacturer’s instructions. Cytokine concentrations were evaluated using Luminex software against standard curves generated with calibration reagents.

### 2.7 Statistical analysis

Data are presented as mean values with standard error of the mean (SEM). Normality of data sets was evaluated by Shapiro Wilk test. If normally distributed, two-group comparisons were performed using two-tailed unpaired t-test. Multi-group comparison was performed using one-way ANOVA. For skewed data sets, non-parametric Mann–Whitney and Kruskal–Wallis tests (followed by Dunns multiple-comparison test) were used. Grouped data sets were analysed using 2-way ANOVA. In multiple tests, Tukey’s, uncorrected Fisher and Dunn’s tests were used to correct *P*-values. All analyses were performed, and graphs created with Prism version 10.0.3 (GraphPad). *P*-values < 0.05 were considered statistically significant.

## 3 Results

All Analyses were performed considering BD activity (active versus inactive BD) and clinical phenotype (mucocutaneous versus ocular BD). Where there was no statistical significance found between these subgroups, data was presented as comparing the complete BD cohort against the healthy control volunteers.

### 3.1 Altered frequency and functional profiles of B-cells and γδT-cells in BD patients with active disease

**Figure 1:**
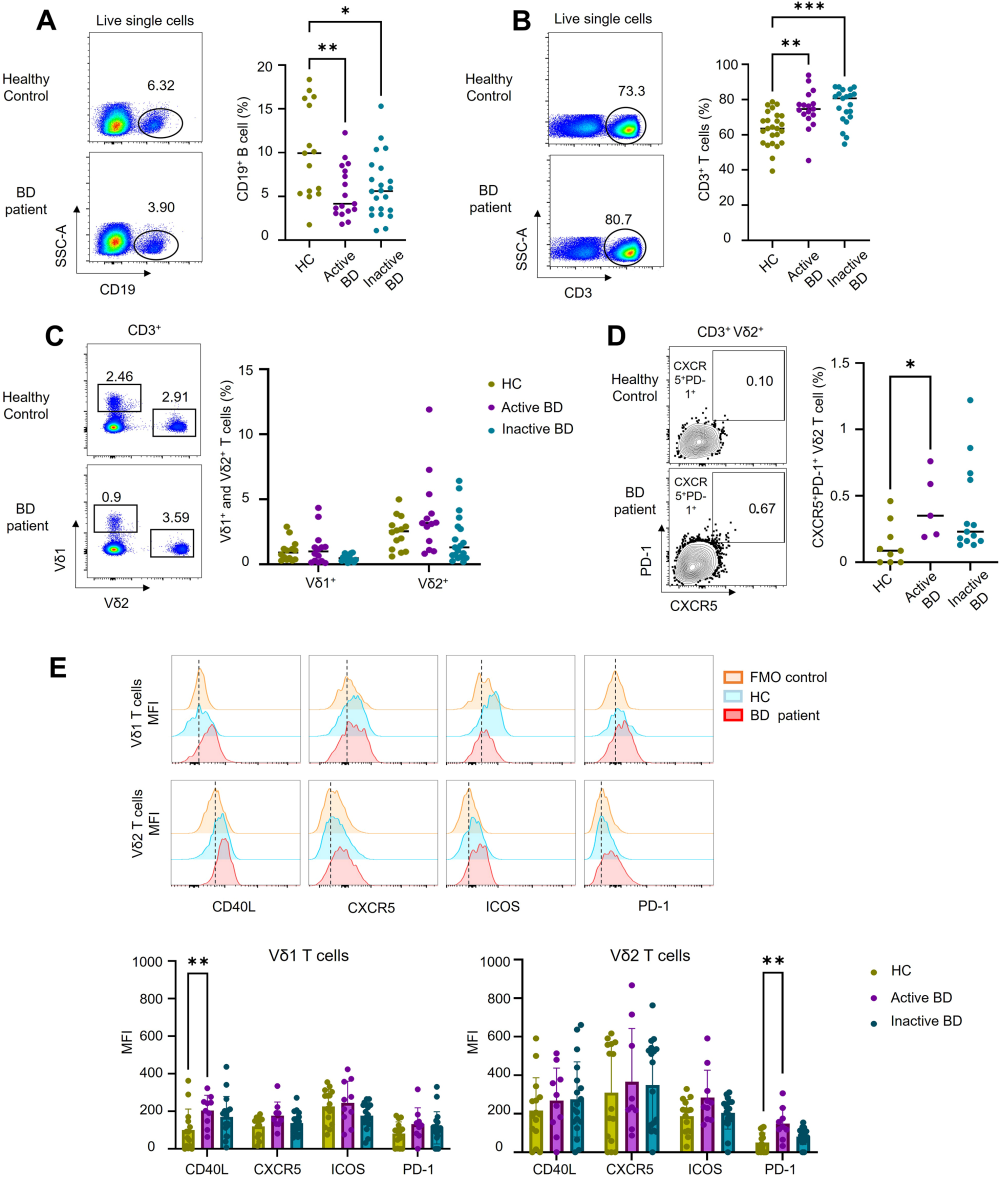
Altered B-cell and γδ T cell profiles in patients with BD Representative flow cytometry plots and cumulative data showing: **(A)** total CD19^+^ B cells (n=15 HC, n=17 active BD, and 22 inactive BD). **(B)** total CD3^+^ T cells (n=25 HC, n=17 active BD, and 21 inactive BD). **(C)** Vδ1 and Vδ2 T cells (n=14 HC, n=13 active BD, and 19 inactive BD). And **(D)** CXCR5+PD-1+ Vδ2 T cells (n= 9 HC, n=5 active BD, and 13 inactive BD). **(E)** Representative histograms and cumulative mean fluorescence intensity (MFI) data showing expression level of CD40L, CXCR5, ICOS and PD-1 in Vδ1 and Vδ2 T cells (n=15 HC, n=10 active BD, and 19 inactive BD). Results show individual values and mean ± SEM. *=*P* < 0.05; **=*P* < 0.01; ***=*P* < 0.001 by Mann-Whitney test and 2-way ANOVA with multiple comparisons. Numbers on plots indicate percentages of cell populations.

Human γδT-cell acquisition of a ‘follicular helper-like’ state has previously been suggested to promote B-cell differentiation (5), but it remains unclear whether this process could contribute to autoantibody generation in BD. To assess this possibility, we first used flow cytometry to profile γδT-cell and B-cell compartments in PBMC from BD patients and HC donors. The total proportion of CD19^+^B cells was significantly reduced in active BD patients (5.5 ± 0.73%) and inactive BD patients (6.05 ± 0.76%) compared to HC (10.11 ± 1.4%; *P* = 0.0061 and 0.01 respectively) (**Figure 1A**). This reduction was more evident in the subgroup of patients with mucocutaneous BD (**Supplementary Figure 1A**).

Next, we investigated γδT-cell profiles within the CD3^+^ pool. The total percentage of CD3^+^T-cells was significantly higher in active BD patients (74.65 ± 2.67%) and inactive BD patients (76.4 ± 2.16%) compared with HC (63.5 ± 1.96%; *P* = 0.0027 and <0.001 respectively) (**Figure 1B**). The frequencies of Vδ1 and Vδ2 T-cells were comparable between healthy and patient groups (**Figure 1C**). Additionally, the total percentage of CD3^+^ and Vδ2T-cells were significantly higher in the subgroup of patients with ocular BD (**Supplementary Figures 1B, C**).

### 3.2 Increased CXCR5^+^PD-1^+^ double positive Vδ2T-cell frequencies in BD patients with active disease

Since data from mouse models has indicated that γδT-cells with a ‘Tfh-like’ profile can license immature B-cells to produce autoantibodies (5), we also analysed γδT-cell expression of key Tfh markers. Indeed, we detected a significantly increased percentage of CXCR5^+^PD-1^+^ double positive Vδ2T-cells in patients with active BD (0.42 ± 0.1%) compared with HC (0.13 ± 0.05%; *P* = 0.0045) (**Figure 1D**). Then we assessed mean fluorescence intensity (MFI) of Tfh markers. Both Vδ1 and Vδ2T cells from BD patients and HC donors showed comparable expression of CXCR5 and ICOS (**Figure 1E**). However, BD patients with active disease displayed significantly higher levels of CD40L expression on Vδ1T-cells (Active BD 204.6 ± 25.47 versus HC 100.6 ± 29.68; *P* = 0.0092) and higher levels of PD-1 expression on Vδ2T-cells (Active BD 148.49 ± 27.59 versus HC 50.33 ± 12.94; *P* = 0.0039) (**Figure 1E**). Altered expression of these markers in BD patients thus appeared to be more closely associated with the disease activity rather than clinical phenotype (**Supplementary Figures 1D, E**).

**Figure 2:**
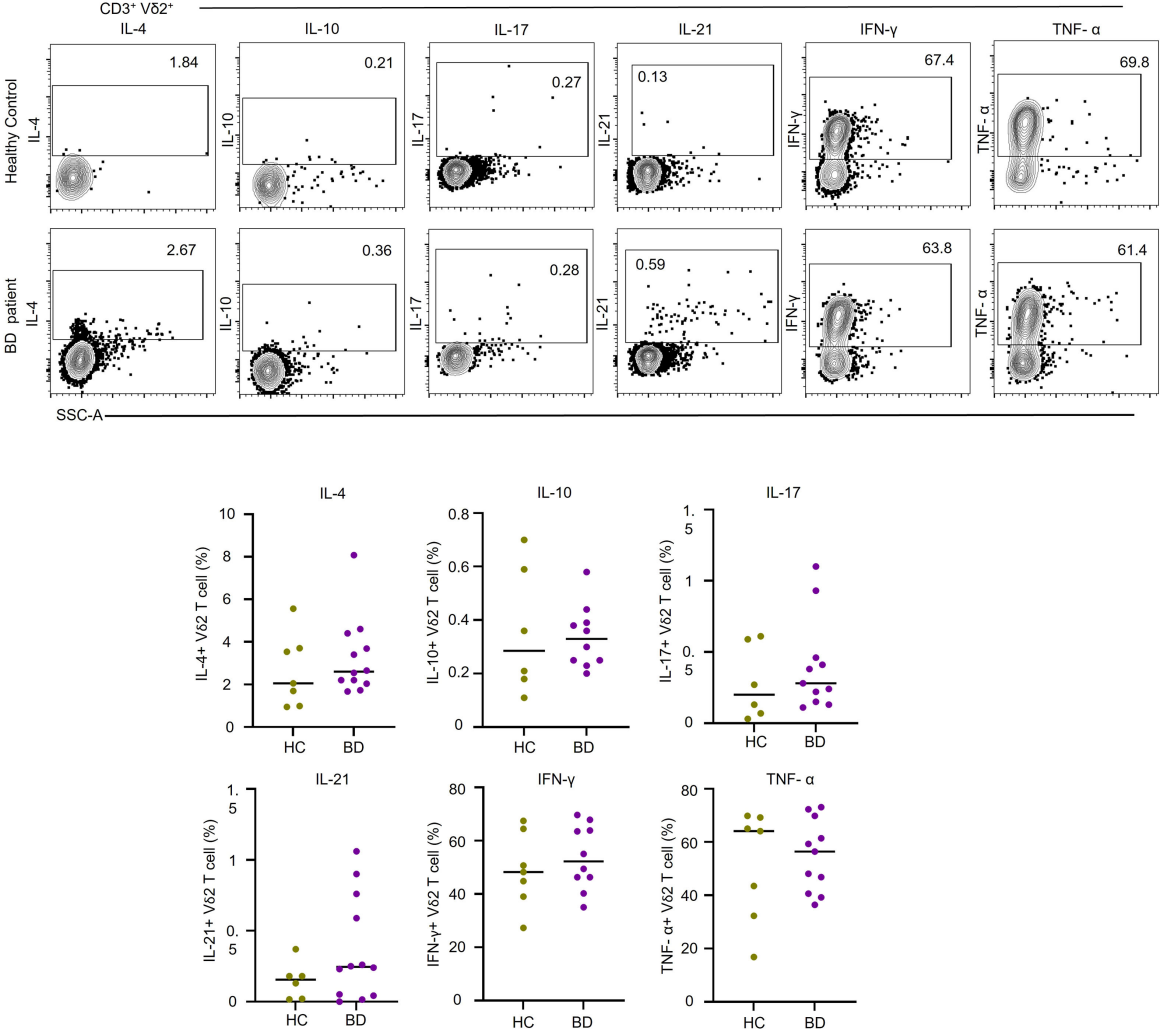
Ex vivo cytokine production by Vδ2 T cells Representative FACS contour plots showing intracellular cytokine expression in PMA-ionomycin-treated Vδ2 T cells. Numbers in plots represent the percentage of cytokine-positive cells. The plot graphs show cumulative data (n=7 HC and n=12 BD patients). Results show individual values and mean frequency.

To further characterize the functional profile of γδT-cells in BD, we assessed *ex vivo* production of six cytokines associated with inflammatory responses and B cell-help (IL-4, IL-10, IL-17, IL-21, IFN-γ, and TNF-α). The levels of these cytokines were comparable between the BD and HC groups in both Vδ2^+^ (**Figure 2**) and Vδ1^+^T-cells (**Supplementary Figure 1F**). Overall, these data show that typical frequencies of B and γδT-cell frequencies are altered in BD, with increased expression of Tfh markers within the γδT-cell compartment in active disease.

### 3.3 Vδ2T Tfh-like cells are enriched in BD and promote B-cell proliferation *in vitro*

**Figure 3:**
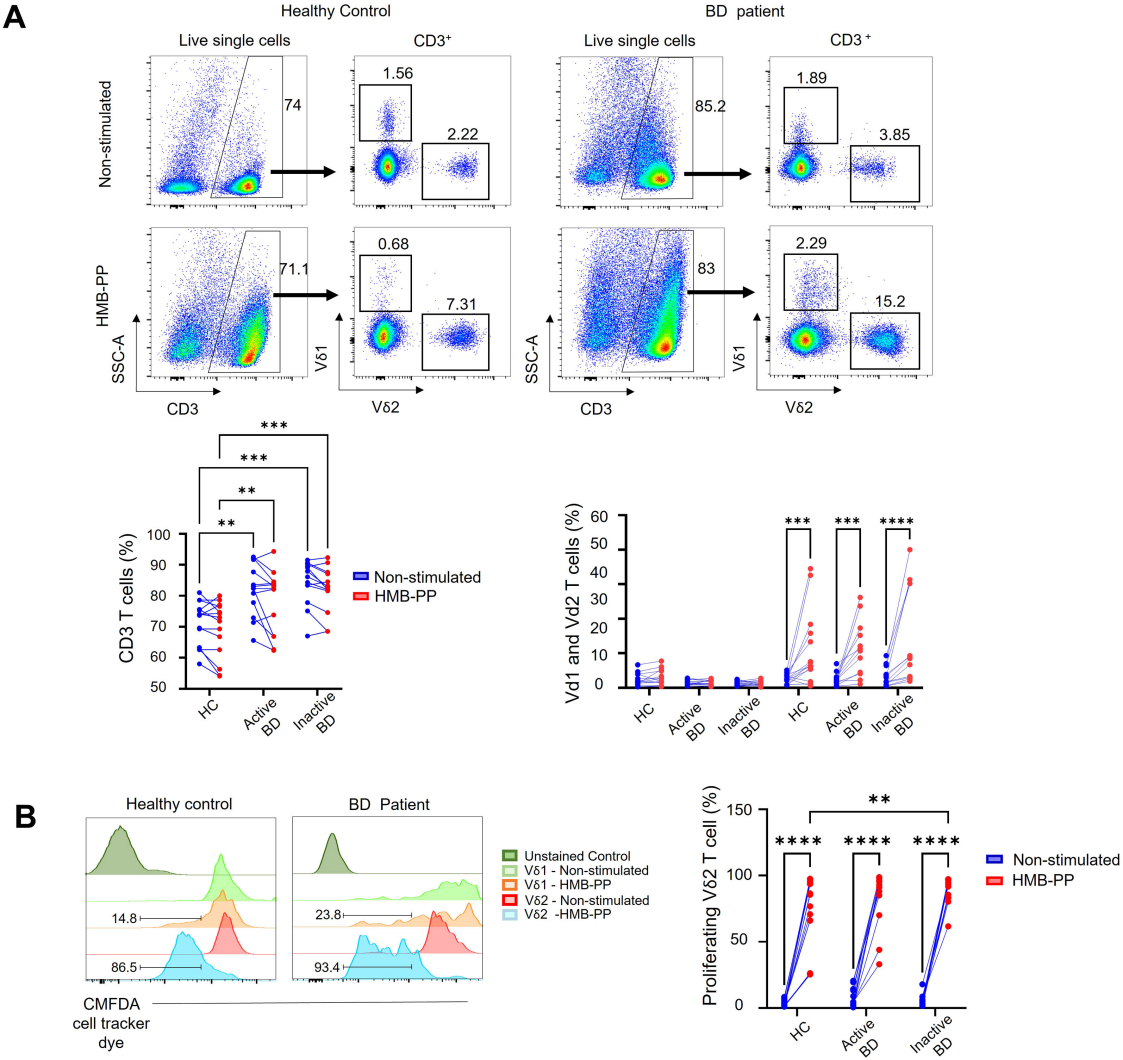
HMB-PP stimulates γδ T cell increased frequency and proliferation in cell culture. **(A)** Representative FACS dot plots and symbol and line graphs showing the frequency of total CD3^+^, Vδ1^+^, and Vδ2^+^ cells in non-stimulated control versus HMB-PP-stimulated PBMC cultures (n=14 HC, n=13 active BD, and 13 inactive BD). **(B)** Representative histograms and symbol and line graphs for CMFDA cell tracker dye signal in Vδ1 and Vδ2 cells as a readout for HMB-PP-induced proliferation in BD patients and HC donors (compared to non-stimulated cells). Numbers on the plots and histograms represent the percentages of cell populations. *=*P* < 0.05; **=*P* < 0.01; ***=*P* < 0.001, ****=*P* < 0.0001 by one and 2-way ANOVA with multiple comparisons.

**Figure 4:**
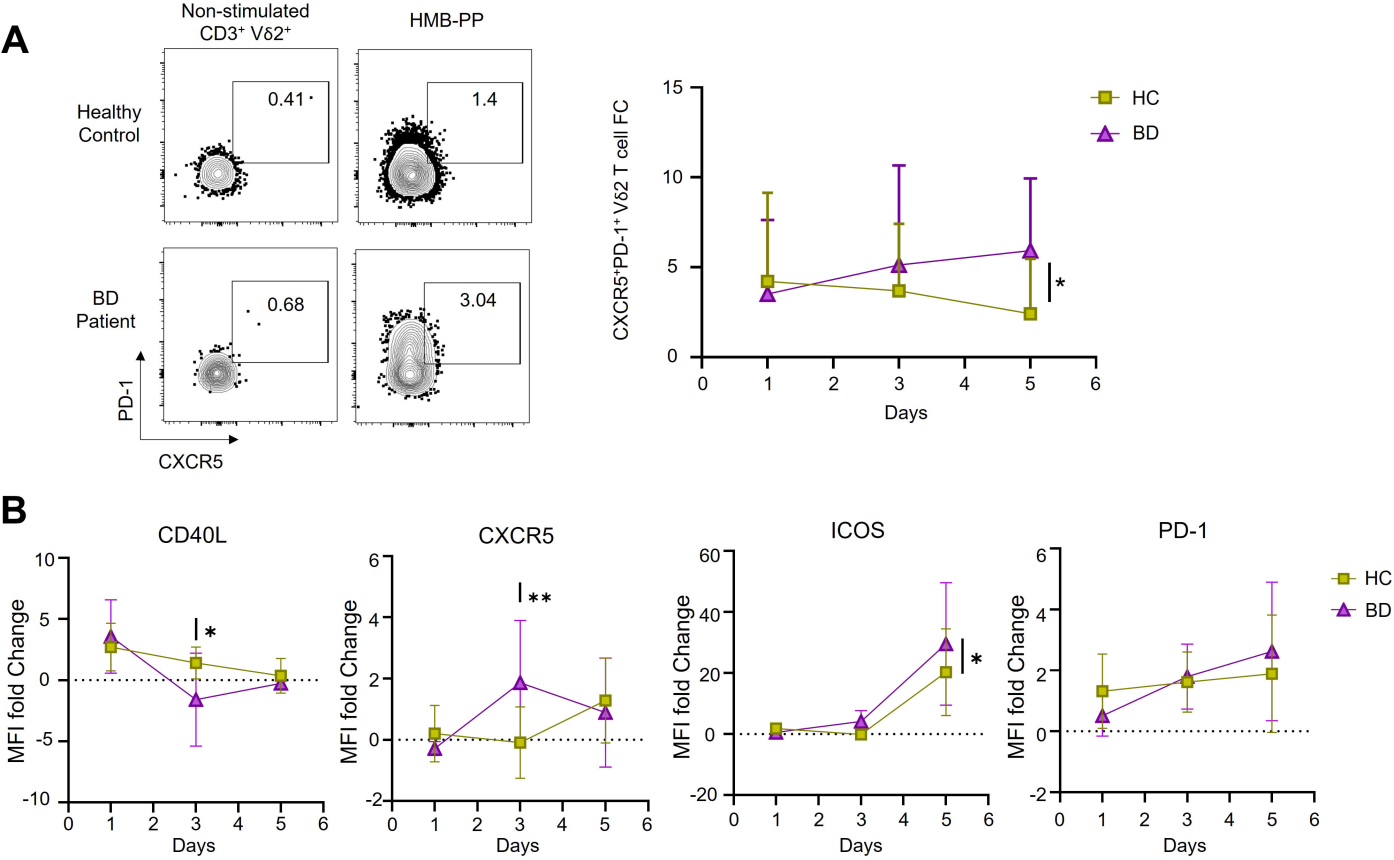
HMB-PP stimulates γδ T cell acquisition of Tfh markers. **(A)** Representative plots showing the frequency and line graph showing fold change of increase of CXCR5^+^PD-1^+^ Vδ2 T cells from either BD or HC donor in non-stimulated control versus HMB-PP-activated PBMC cultures on day 5. **(D)** Line graphs showing fold-changes in Tfh marker MFI by Vδ2T cells at different time points during culture of PBMC from HC donors or patients with BD (n=27 BD, n=11 HC). Numbers on the plots and histograms represent the percentages of cell populations. Panels show the mean ± SEM of MFI for Tfh markers. *=*P* < 0.05; **=*P* < 0.01; by one and 2-way ANOVA with multiple comparisons.

To investigate the possible functional effects of Vδ2^+^ cells with a Tfh-like functional profile in BD, we next used microbial metabolite HMB-PP to selectively activate Vδ2^+^ T cells in total PBMC cultures and observed the impact on B-cell activation and differentiation over 5 days. As expected, total CD3^+^T cell numbers were not substantially altered after 5 days stimulation with HMB-PP (**Figure 3A**) whereas the Vδ2^+^ subset was selectively expanded in BD patients (Active BD; non-stimulated 2.53 ± 0.52% versus HMB-PP stimulated 11.4 ± 2.3%, *P* < 0.001) (Inactive BD; non-stimulated 3.32 ± 0.82% versus HMB-PP stimulated 14.64 ± 4%, *P* < 0.001), and HC donors (non-stimulated 2.6 ± 0.4% versus HMB-PP stimulated 11.2 ± 2.9%; *P* < 0.001) (**Figures 3A, B**). Additionally, CXCR5^+^PD-1^+^ Vδ2T-cell frequency fold change (calculated of non-stimulated and HMB-PP-stimulated cultures at each time point) increased over time in the BD group while decreasing in HC, with a significant difference observed by day 5 (BD: 5.9 ± 0.8% versus HC: 2.4 ± 1%, *P* = 0.04), (**Figure 4A**). This expansion was accompanied by dynamic changes in expression of selected Tfh markers, presented here as MFI fold change of increase between stimulated and non-stimulated conditions for each sample at each time point and presented as compared means. In the BD group, CD40L expression fold change decreased markedly by day 3 (*P* = 0.02) but recovered to levels comparable level with HC samples by day 5. In contrast, CXCR5 expression fold change peaked at day 3 in BD patients, which was significantly higher than in HC samples (*P* = 0.008). ICOS and PD-1 expression fold changes increased over time in both groups, reaching significantly higher values of ICOS in the BD group by day 5 (*P* = 0.04) (**Figure 4B**). These findings suggest altered dynamics of Tfh-like functional state acquisition by Vδ2T-cells from BD patients.

**Figure 5:**
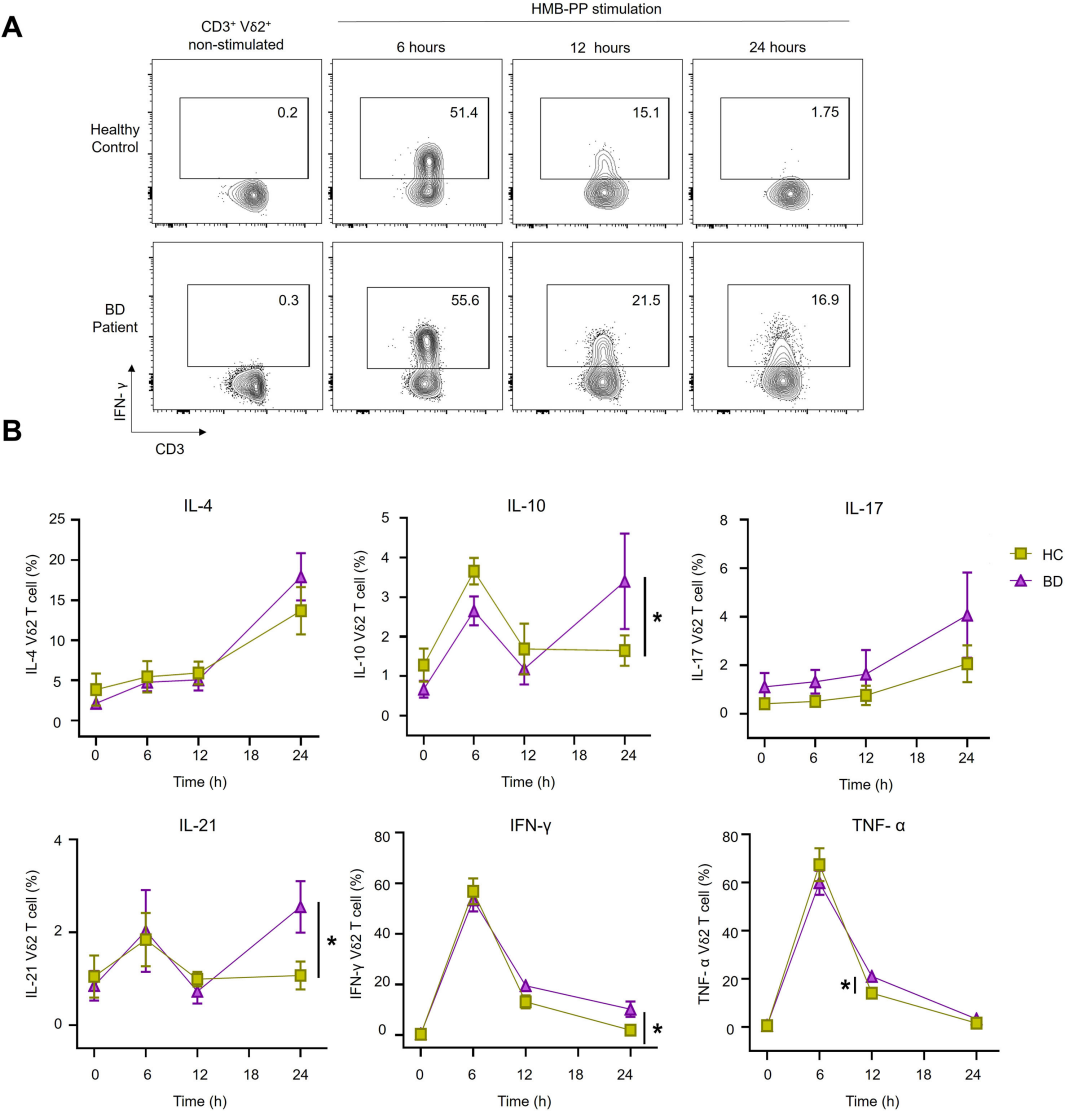
Distinct patterns of intracellular cytokine production by Vδ2 T cells following HMB-PP- stimulation. **(A)** Representative FACS contour plots showing intracellular IFN-γ expression in stimulated Vδ2 T cells from either HC and BD patient at 0 (unstimulated), 6, 12, and 24 hours post-HMB-PP stimulation. **(B)** Line graphs showing frequency of Vδ2 T cells producing IL-4, IL-10, IL-17, IL-21, IFN-γ, and TNF-α (HC = 7, BD = 12). Numbers on plots represent the percentages of cell populations. Data in line graphs represent the mean ± SEM %. *=P<0.05; by 2-way ANOVA tests with multiple comparisons.

We next assessed the cytokine profiles of Vδ2T-cells after 6, 12, and 24 hours of HMB-PP stimulation compared with non-stimulated cells (**Figure 5**). Distinct expression patterns emerged over time. For instance, IL-4^+^ and IL-17^+^ cells tended to rise over time in both groups. In contrast, IFN-γ ^+^ and TNF-α ^+^ cells peaked at 6 hours in both groups and then decreased gradually, maintaining a significantly higher level of expression in BD patients (IFN-γ^+^ cells; BD: 10.1 ± 3% versus HC: 1.8 ± 0.3%, *P* = 0.03), (TNF-α^+^ cells; BD: 21 ± 1.9% versus HC: 14 ± 2.1%, *P* = 0.038). Interestingly, IL-10 and IL-21 also peaked at 6 hours but was maintained at significantly higher levels in the BD group by 24 hours (IL-10^+^ cells; BD: 3.4 ± 1.2% versus HC: 1.64 ± 0.38%, *P* = 0.03) (IL-21^+^ cells; BD: 2.54 ± 0.55% versus HC: 1.07 ± 0.3%, *P* = 0.04) (**Figure 5**).

### 3.4 Activated Vδ2 cells with Tfh-like functional profile support B cell transition to a plasma cell profile in BD

**Figure 6:**
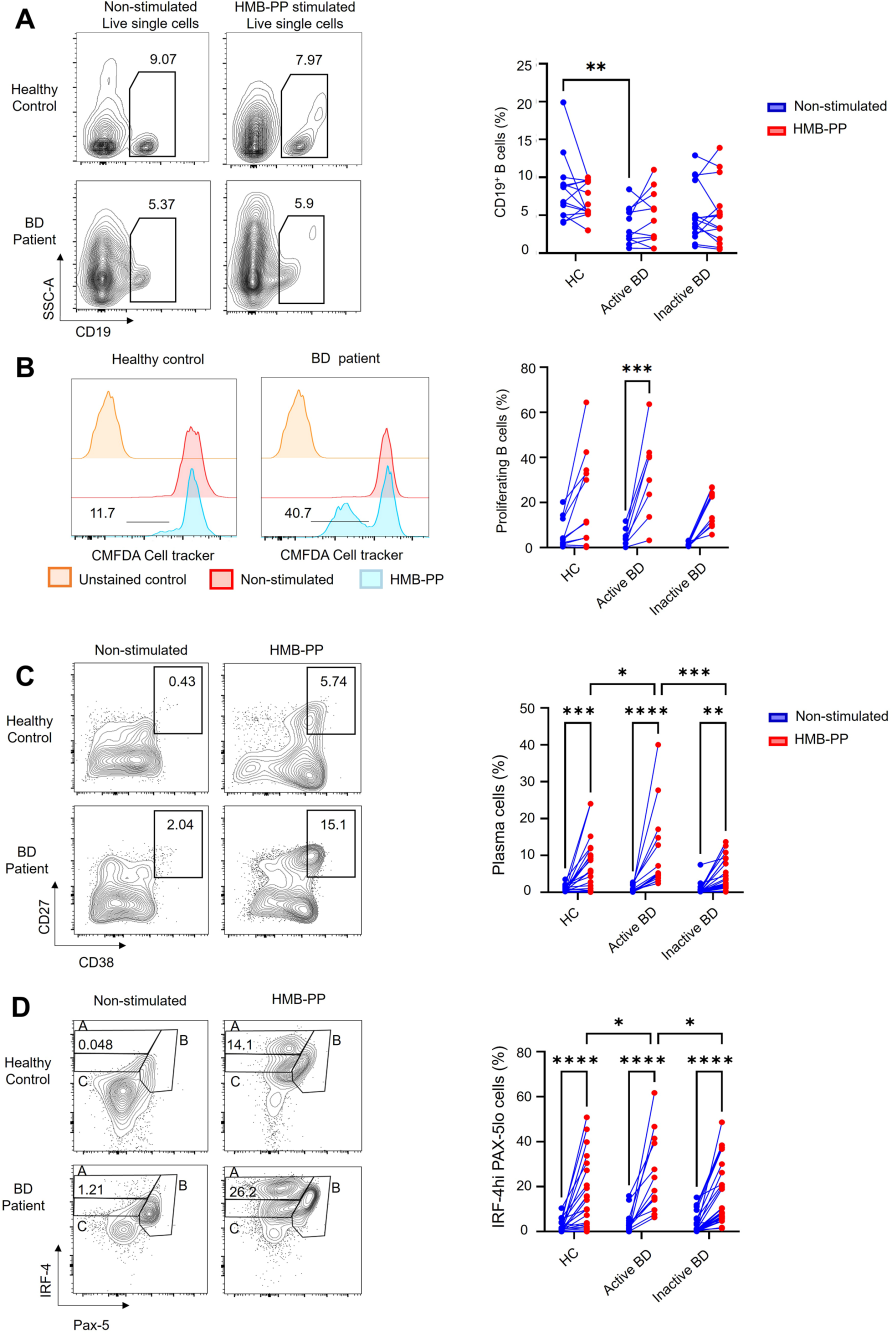
Activated Vδ2T-cells induce B-cell proliferation and differentiation into autoantibody-producing plasma cells. **(A)** Representative FACS plots and cumulative data showing total CD19^+^B cells in control versus HMB-PP-activated PBMC cultures on day 5 (n=14 HC, n=13 active BD, and 13 inactive BD). **(B)** Representative histograms and symbol and line plots of CMFDA cell tracker dye signal in B cell proliferation after HMB-PP stimulation in HC and BD patients compared to non-stimulated cells. **(C)** Representative FACS plots and graphs showing frequency in induced plasma cells (CD19^+^CD27^+^CD38^hi^) after 5 days in control versus HMB-PP-stimulated cultures (n= 24 HC donors, n=14 active disease, and 31 inactive BD). **(D)** Representative FACS plots and graphs showing frequency in CD19^+^IFR4^hi^PAX5^lo^ cells on day 5 in control versus HMB-PP-stimulated cultures of PBMC. Data represent the mean ± SEM %. Numbers on plots and histograms represent the percentages of cell populations. *=*P* < 0.05; **=*P* < 0.01; ***=*P* < 0.001, ****=*P* < 0.0001 by 2-way ANOVA and multiple comparisons.

We next investigated the ability of activated Vδ2T-cells to induce B cell proliferation and differentiation *in vitro*. First, we established a positive control culture system to confirm efficient B cell proliferation and differentiation under our culture conditions (**Supplementary Figure 2**). Next, we tested the effect of HMB-PP-activated Vδ2T-cells on B-cell proliferation and differentiation within total PBMC cell cultures. While the total proportions of CD19^+^B cells were similar in BD and HC groups following stimulation (**Figure 6A**), the frequency of proliferating B-cells detected was significantly higher in BD patients with active disease (4.6 ± 1.3% versus 32.1 ± 6.6%, *P* < 0.001), compared to both inactive BD (1.8 ± 0.3% versus 17.4 ± 2.5%, *P* = 0.06), and HC donors (7 ± 2% versus 21.5 ± 6.2%, *P* = 0.08), (**Figure 6B**).

**Figure 7:**
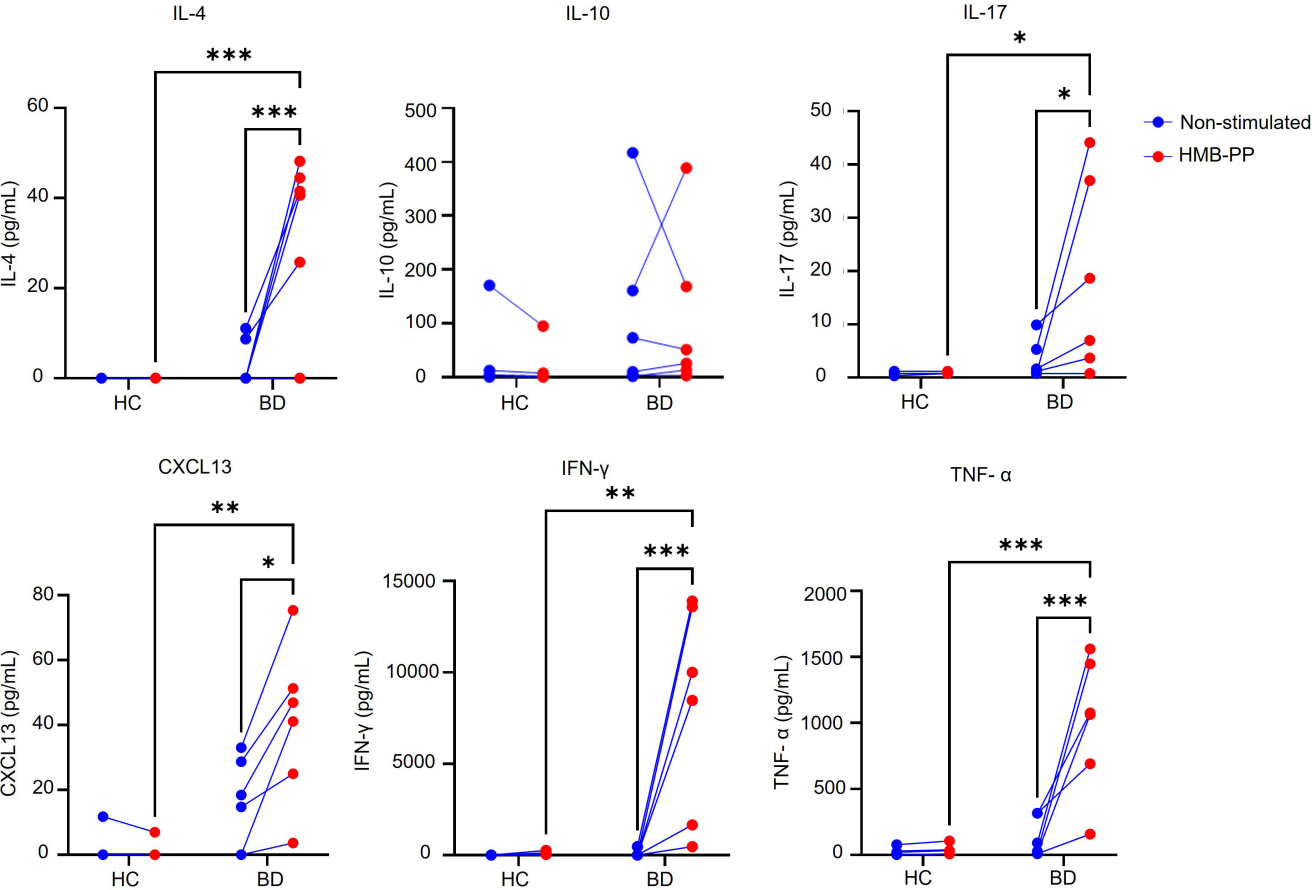
Vδ2T-cell activation induces a cytokine milieu associated with B-cell differentiation. Symbol and line graphs showing cytokine levels in cell culture supernatant measured after 1 day of HMB-PP stimulation (HC = 4, BD = 6) relative to unstimulated control cultures. Figures show individual values. *=*P* < 0.05; **=*P* < 0.01; ***=*P* < 0.001 by 2-way ANOVA with multiple comparisons.

Using the same culture system, we next investigated whether increased frequency of proliferating B-cells was linked with enhanced differentiation into plasma cells (CD19^+^CD27^+^CD38^hi^). Following HMB-PP stimulation, the percentages of plasma cells were significantly higher in the Active BD group (10.85 ± 2.95%) compared to the inactive BD (4.2 ± 0.76%, *P* = 0.02) and HC groups (6.4 ± 1.45%, *P* < 0.001) (**Figure 6C**). Since expression of IRF-4 and PAX-5 in CD19^+^B-cells controls differentiation into plasma cells, we proceeded to test whether HMB-PP activation of Vδ2T cells could influence the expression of these transcription factors. Flow cytometry analyses showed that under HMB-PP-stimulated conditions, the frequency of IFR-4^hi^PAX-5^lo^ cells was significantly higher in the Active BD group (25.13 ± 4.77%) compared to the inactive BD (15.14 ± 2.51%, *P* = 0.038) and HC (15.81 ± 3.18%, *P* = 0.19) (**Figure 6D**), indicating enrichment of a subset reported to display the highest potential for differentiation into antibody-producing plasma cells (31). Since changes in B-cell expression of IRF-4 and Pax-5 might be associated with the cytokine milieu generated by HMB-PP stimulation in total PBMC cell cultures, we next quantified supernatant levels of key cytokines and chemokines at early (**Figure 7**) and late (**Supplementary Figure 3**) timepoints of the cell cultures (on day 1 and 5 of culture). Using the Luminex assay to assess multiple cytokines and chemokines in cell culture supernatants (IL-4, IL-10, IL-17, IL-21, CXCL13, IFN-γ, TNF-α), we observed that all mediators apart from IL-10 were expressed at higher levels in the BD group, whereas IL-21 could not be detected in day 1 cultures (**Figure 7**). These early differences tended to resolve with increasing duration of culture, such that measured cytokine levels were comparable between BD and HC cultures by day 5 (**Supplementary Figure 3**; **Supplementary Table 7**).

### 3.5 Anti-HSP60 autoantibodies are increased in patients with BD

**Figure 8:**
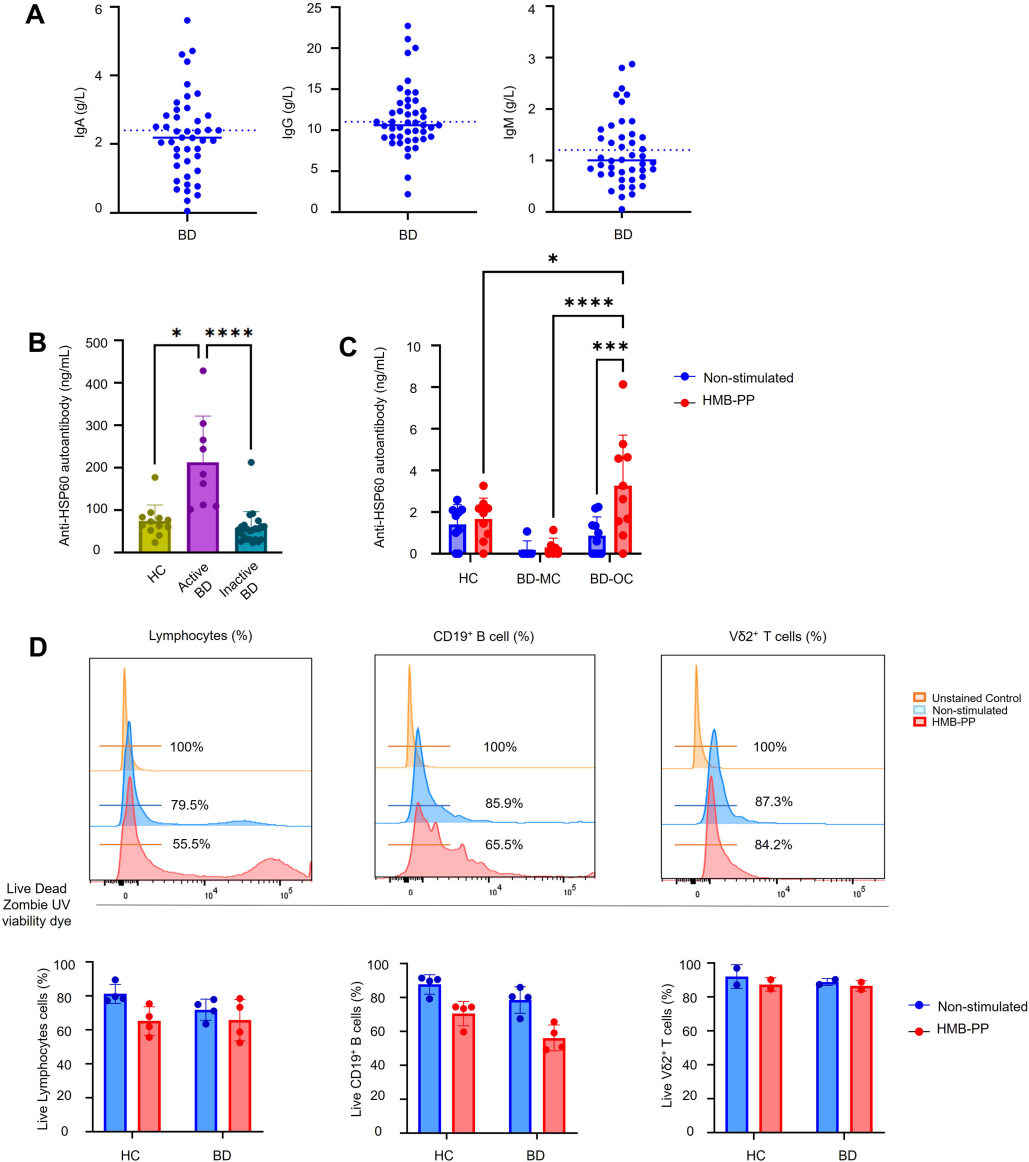
Increased levels of anti-HSP60 autoantibodies in patients with BD. **(A)** Total concentration of IgA, IgG, and IgM antibodies in blood serum from BD patients (n=44). Dotted lines indicate the average concentration reported for healthy adults. Concentration of anti-HSP60 autoantibodies (IgA, IgG and IgM) in **(B)** BD and HC serum (HC = 12, Active BD = 9, Inactive BD = 23), or **(C)** cell culture supernatants (HC = 9, mucocutaneous BD = 6, and ocular BD = 10). **(D)** Representative histograms and cumulative data of cell viability in bar charts showing frequencies of live lymphocytes, CD19^+^ B cells and Vδ2^+^ T cells after 10 days of cell culture. Plots show individual values and mean. *=*P* < 0.05; **=*P* < 0.01; ***=*P* < 0.001, ****=*P* < 0.0001 by Mann Whitney’s, Kruskal-Wallis’s, and 2-way ANOVA tests with multiple comparisons.

Given that BD disease activity correlated with Vδ2 Tfh-like functional profile and B cell function across multiple assays, we next used clinical data to explore potential associations with serum levels of IgA, IgG, and IgM. Immunoglobulin concentrations in BD patients were similar to reported healthy adults mean levels and within normal range (**Figure 8A**). However, anti-HSP60 autoantibody levels were significantly higher in serum from patients with active BD (212.5 ± 36.4ng/mL) compared with inactive disease (57.65 ± 8.1 ng/mL; *P* = 0.0001) or HC donors (74.36 ± 10.9 ng/mL; *P* = 0.013) (**Figure 8B**). Independent from disease activity, supernatant levels of anti-HSP60 autoantibodies were significantly increased after HMB-PP stimulation of cell cultures of BD patients with ocular phenotype (3.25 ± 0.77 ng/mL) compared to mucocutaneous (0.3 ± 0.18 ng/mL; *P* < 0.0001) and HC donor cells (1.66 ± 0.33 ng/mL, *P* = 0.012) (**Figure 8C**). In the long-term cell cultures, cell viability remained high at day 10 when harvesting the supernatants for the functional autoantibody readout (**Figure 8D**).

## 4 Discussion

BD pathogenesis is associated with abnormal frequency and activation of major lymphocyte lineages (1, 2) including polyclonal B-cell activation (3), and spontaneous antibody production (2), but the underlying disease mechanisms remain unclear. A key role for B-lymphocytes has been demonstrated by significant clinical improvement in BD symptoms following B-cell depletion using anti-CD20 antibodies (33, 34). We now provide evidence that stimulation of circulating Vδ2T cells within an intact PBMC context can promote B-cell differentiation and exert an important influence on systemic autoantibody production in patients with BD and could therefore represent an additional cellular target for novel therapies.

We observed higher blood Vδ2T-cell frequencies in active BD, consistent with the findings of other investigators reporting higher frequencies in BD in general (23, 35). However, it is important to note that some previous studies have identified comparable γδT-cell numbers in BD and healthy adults (36), potentially due to the confounding effects of variable disease activity, immunosuppressive medications, and/or co-morbidities. Nonetheless, there is evidence that γδT-cells can regulate B cell functions via different mechanisms, including expression of Tfh markers (CD40L, CXCR5, ICOS, and PD-1) and production of cytokines (IL-4, IL-10, and IL-21) required to prime antibody production (10, 37–40). In active BD, we detected elevated PD-1 expression by Vδ2T-cells, perhaps indicating ongoing activation of this subset. Additionally, we detected higher frequencies of CXCR5^+^PD-1^+^ double positive Vδ2T-cells in patients with active BD, which resembles the classical functional definition of Tfh CD4^+^ cells. Together, these data suggest that Vδ2T-cells can acquire a non-classical ‘Tfh-like’ functional state in BD patients, consistent with the known functional flexibility of Vδ2T cells in autoimmune conditions (10, 41). The Vδ2T and B cell interactions within the context of total PBMCs have been studied in human and mice studies, because this approach preserves the physiological multicellular context in which Vδ2 T cells and B cells normally interact (28–30). However, it’s important to understand the direct and indirect axis of this interaction. Caccamo, et.al, successfully isolated circulating Vδ2 T cells and tonsillar B cells from healthy donors and demonstrated, using a co-culture system, that HMB-PP stimulation replicated the effects noted in the total PBMC cultures (10, 37–40); these results suggest the possibility of a direct interaction between the 2 isolated populations independent of other cell types. Nonetheless, a multicellular context should be considered especially under immune dysregulation conditions like in BD. Multiple additional cells might be involved in mediating interactions between Vδ2 T and B cell interactions, including antigen presenting cells such as dendritic cells, classical CD4 Tfh cells, or cytokine producer cells such as monocytes (10, 37, 40).

Classical interactions between Tfh and B cells are reported to occur around 1–2 days after exposure to antigen (39). Liu et al. observed that CD40L is upregulated on activated CD4 T-cells as early as 6–24 hours following antigen recognition, then declines gradually in parallel with ICOS and CXCR5 upregulation from 48–72 hours (39). Shi et al. demonstrated that ICOS can then promote expression of CXCR5 and PD-1 (40), which aligns with our current findings. Here, we observed that CD40L expression decreased significantly after the first day of stimulation in BD patients accompanied by marked increases in both intracellular and released levels of IL-4, IL-10, IL-17, IL-21, IFN-γ, TNF-α, and B-cell-attracting chemokine CXCL13. Furthermore, from culture day 3 onwards both ICOS and CXCR5 were markedly induced in BD cells but not HC cultures. These data suggest that while the Vδ2T subset displays a similar proliferative response to HMB-PP in both BD and HC donors, as expected (37), these cells acquire a more prominent Tfh-like functional state in BD early after stimulation, resembling the functional profile of circulating Tfh cells in humans (13, 14), rather than the classical Tfh cells located in germinal centres. This might imply a functional divergence or lack of regulatory mechanisms that control expression of these markers and cytokines in HC cells. These findings are in-line with previous reports that γδT-cells can express key Tfh markers and B-cell-helping cytokines in both mice and humans (10, 37, 38). There is also evidence that abnormal Vδ2T-cell crosstalk with B cells can lead to overproduction of IL-4, potentially breaking tolerance and leading to polyclonal B-cell responses and autoantibody production (5, 42).

It has recently become clear that γδT-cell activation in both mice and humans can exert a strong impact on humoral immunity under steady-state conditions (5, 9), but also in pathological settings such as systemic lupus erythematosus (SLE) (10, 43). We observed that Vδ2T-cell activation in our PBMC culture system was associated with significantly increased B-cell proliferation in BD patients relative to HC donors. B cell proliferation and differentiation into plasma cells are known to be regulated by nuclear transcription factors including Blimp-1, IRF4, and Pax5 (44, 45). Once committed to differentiation, B cells first increase Blimp-1 expression, which then regulates PAX5-mediated maturation, whilst IRF-4 supports the survival of mature plasma cells (31). Our analysis showed that plasma cell induction and IRF-4^hi^Pax-5^lo^ cell frequency were significantly higher in HMB-PP-stimulated cultures from BD patients with active disease. This suggests that help provided by specifically stimulated Vδ2T-cells might promote B cell activation in total PBMC context, breaking of tolerance, and support autoantibody production. In mice with severe combined immunodeficiency (SCID), adoptive transfer of γδT with B cells is sufficient to induce germinal centre development, providing *in vivo* evidence that γδT-cells alone can regulate B cell follicular responses (46). These γδT-cell-dependent germinal centres have also been reported to display high rates of class-switching and autoreactive antibody generation (37, 47). Consistent with these animal data, human γδT cell lines have also been shown to induce rapid autoantibody production during co-culture with autologous B cells (11, 37) via mechanisms that involve ICOS, CD40L, IL-4, IL-10, and IL-21 (37, 48). These other mediators have already been strongly linked with BD disease activity (12, 49), and effective treatment options include blocking or neutralising antibodies against key pro-inflammatory cytokine TNF-α (50). Bacterial metabolites are potent inducers of IFN-γ expression in human mucosal γδT-cells (51), which may also be triggered by infection in the context of BD (52), whereas non-specific activation of this compartment could lead to increased production of IL-17 (41).

The role of immunoglobulins in BD pathogenesis remains controversial (2). Resembling the findings of several other studies (19, 53, 54), our analysis of anti-HSP60 autoantibody levels indicated significantly higher concentrations in the serum of BD patients with active disease. Previous reports stated that selectively activated Vδ2T-cell displaying high expression of CXCR5, IL-10, and IL-21 can support antibody production *in vitro* (10, 37). Consistent with these data, we also detected increased levels of anti-HSP60 autoantibodies in our extended BD cell cultures stimulated with HMB-PP. Human HSP60 is upregulated in inflamed ocular tissues and active oral ulcers in BD (20, 21). Additionally, high titres of anti-retinal HSP60 have previously been correlated with BD-associated uveitis in human studies and mouse models (19, 53). Considering that γδT-cells can recognise mycobacterial and streptococcal-derived HSP65 (2, 21–23) a close molecular mimic of human HSP60, and that such recognition might induce host responses against human HSP60 (24, 25), thereby promoting autoantibody generation in BD (26), we selected the bacterial antigen HMB-PP as a well-documented bacterial stimulus for Vδ2 T cells. This approach allowed us to evaluate the B-cell–helping function of Vδ2 T cells, which act as in an MHC-independent and non-antigen-specific manner. Our results therefore suggest that Vδ2T-cell-mediated activation of B cells -within a multicellular PBMC context- may promote the generation of anti-HSP60 autoantibodies, which may in-turn be associated with distinct BD phenotypes and disease activity (19, 55). Importantly, these data extends the evidence that Vδ2T-cells can provide broad B-cell-help -with possible contribution of other immune cells- and enhance non-specific autoantibody production following activation with an unrelated antigen such as HMB-PP rather than HSP60 protein itself (37).

We acknowledge certain limitations in our study. The blood sample volumes collected from the patients were limited - in accordance with the study’s ethical approval conditions, thus restricting replicating the same patient cohort across all our cultures and assays. As a tertiary referral center, all BD patients recruited at the BD centre of Excellence - are currently receiving treatment, varying from topical creams up to immunosuppressive biological options. We conducted our cultures using total PBMCs by design to mimic physiological *in vivo* conditions (28–30). Further studies using sorted Vδ2T and B cell cultures will help answer the question about indirect help from other immune cells. Also, our work could be complemented by biopsy-focused studies to assess whether similar B-helper features are highlighted in tissue Vδ2T cells. Transcriptomic analysis will help uncover the direct mechanism by which Vδ2T-cells promote autoantibody production in BD.

## 5 Conclusions

Many autoimmune diseases are characterized by marked production of autoantibodies, typically thought to be supported by Tfh cells *in vivo*. The current study provides new evidence that alternative Vδ2T cell functional profiles may also provide effective help for autoantibody production in the context of BD. Identifying the exact mechanisms and mediators underpinning this interaction will improve current understanding of BD pathophysiology and could potentially lead to the development of novel therapies for affected patients.

## Data Availability

The raw data supporting the conclusions of this article will be made available by the authors, without undue reservation.

## Supplementary material

Supplementary Figure 1 Profiling of CD19^+^ B-cells and γδ T cells *ex vivo* across BD clinical phenotypes The dot plot shows the frequency of total CD19^+^B cells **(A)** total CD3^+^ T cells **(B)**, and γδ T cells **(C)** in HC donors and patients with BD grouped according to clinical phenotype (mucocutaneous MC, or ocular OC) (n=25 HC, 30 BD-MC, and 28 BD-OC). Cumulative mean fluorescence intensity (MFI) data for Vδ1+ **(D)** and Vδ2+ populations **(E)** (n=15 HC, 16 BD-MC, and 13 BD-OC). F) Summary dot plots showing frequency of Vδ1 T cells producing IL-4, IL-10, IL-17, IL-21, IFN-γ, and TNF-α (n=7 HC and n=12 BD patients). Results show individual values and mean ± SEM. *=*P* < 0.05; **=*P* < 0.01; ***=*P* < 0.001, ****=*P* < 0.0001 by Mann Whitney test, one and 2-way ANOVA with multiple comparisons.

Supplementary Figure 2 Positive control B cell simulation during *in vitro* culture **(A)** B cell proliferation after direct stimulation in HC donors and BD patients compared to non-stimulated cells is shown in representative histograms and as individual values in symbol and line plots. Frequency of induced plasma cell (CD19^+^CD27^+^CD38^hi^) and IRF-4^hi^PAX-5^lo^ CD19^+^B cells in non-stimulated versus direct B cell stimulation is shown in representative flow cytometry plots and in symbol and line plots (**(B, C)** respectively). Numbers on the plots and histograms represent the percentages of cell populations. *=*P* < 0.05; **=*P* < 0.01; ***=*P* < 0.001, ****=*P* < 0.0001 by 2-way ANOVA and multiple comparisons.

Supplementary Figure 3 Changes in cytokine levels after prolonged Vδ2T-cell activation Symbol and line graphs showing cytokine levels in cell culture supernatant measured after 5 days of HMB-PP stimulation in HC donors (n=11) and BD patients (n=25) relative to unstimulated control cultures. Figures show individual values. *=*P* < 0.05; **=*P* < 0.01; by 2-way ANOVA with multiple comparisons.

Supplementary Table 1 Flow-cytometry panel for analysis of B-cell and γδ T-cell subpopulations.

Supplementary Table 2 Flow-cytometry panel for analysis of B-cell and γδTfh-like profiles.

Supplementary Table 3 Flow-cytometry panel for analysis of cultured B-cells and γδ cells.

Supplementary Table 4 Flow cytometry panel for analysis of plasma cells and transcription factor expression.

Supplementary Table 5 Flow-cytometry panel for analysis of intracellular cytokines in γδ+T cells.

Supplementary Table 6 Flow-cytometry panel for analysis of intracellular cytokines in γδ+T cells.

Supplementary Table 7 Multiplex Cytokine array results (Mean ± SEM) and fold change of increase after HMB-PP stimulation.
