# Supplementary figures and images for: Follicular helper-like γδ T cells promote plasma cell differentiation in Behçet’s disease
Source: Front Immunol. 2026 Feb 9;17:1763174. doi: 10.3389/fimmu.2026.1763174 (PMC12926121; doi:10.3389/fimmu.2026.1763174)

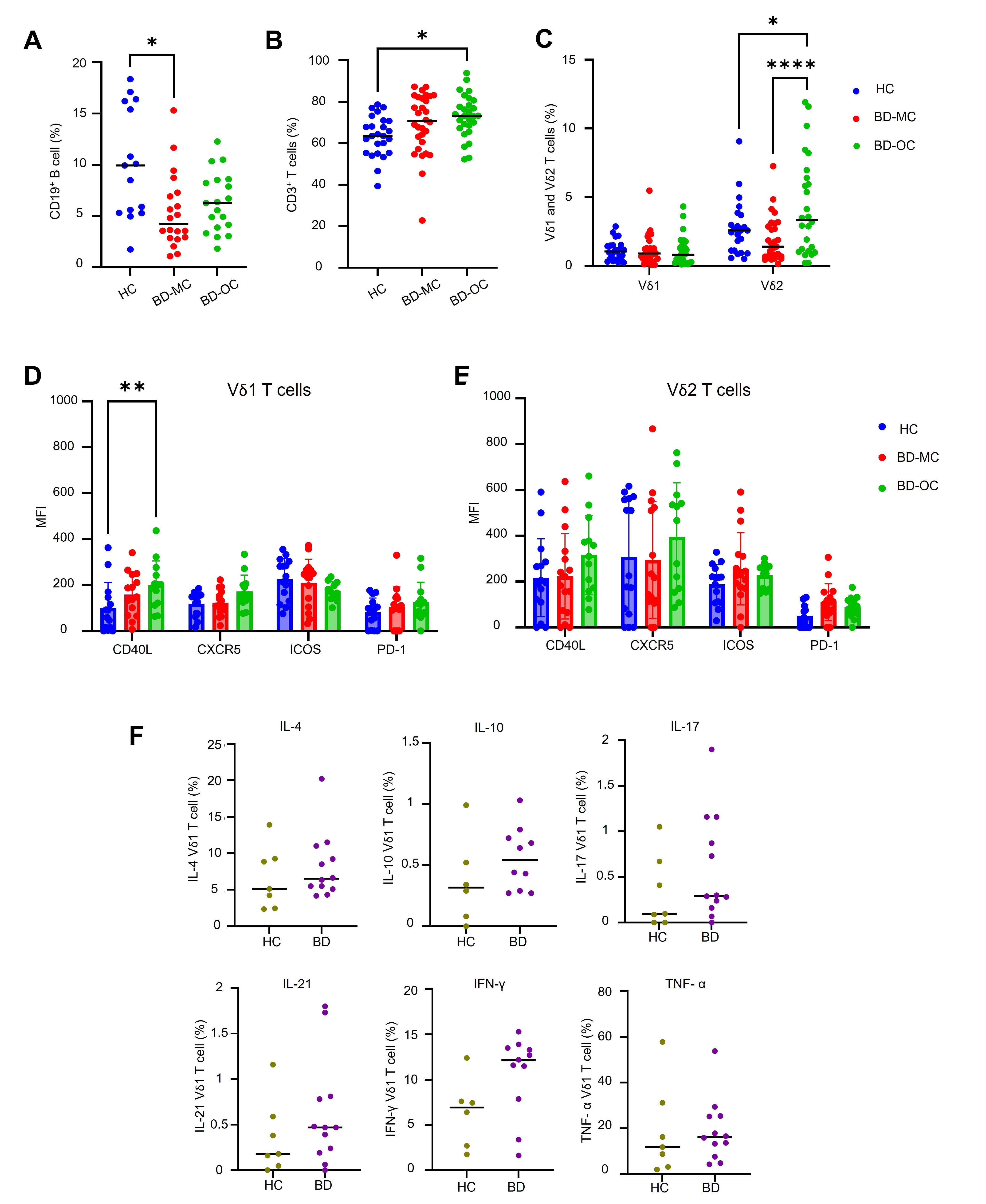

Supplement: Supplementary Figure 1 — Profiling of CD19+ B-cells and γδ T cells ex vivo across BD clinical phenotypes The dot plot shows the frequency of total CD19+B cells (A) total CD3+ T cells (B), and γδ T cells (C) in HC donors and patients with BD grouped according to clinical phenotype (mucocutaneous MC, or ocular OC) (n=25 HC, 30 BD-MC, and 28 BD-OC). Cumulative mean fluorescence intensity (MFI) data for Vδ1+ (D) and Vδ2+ populations (E) (n=15 HC, 16 BD-MC, and 13 BD-OC). F) Summary dot plots showing frequency of Vδ1 T cells producing IL-4, IL-10, IL-17, IL-21, IFN-γ, and TNF-α (n=7 HC and n=12 BD patients). Results show individual values and mean ± SEM. *=P < 0.05; **=P < 0.01; ***=P < 0.001, ****=P < 0.0001 by Mann Whitney test, one and 2-way ANOVA with multiple comparisons. [file Image1.jpeg]

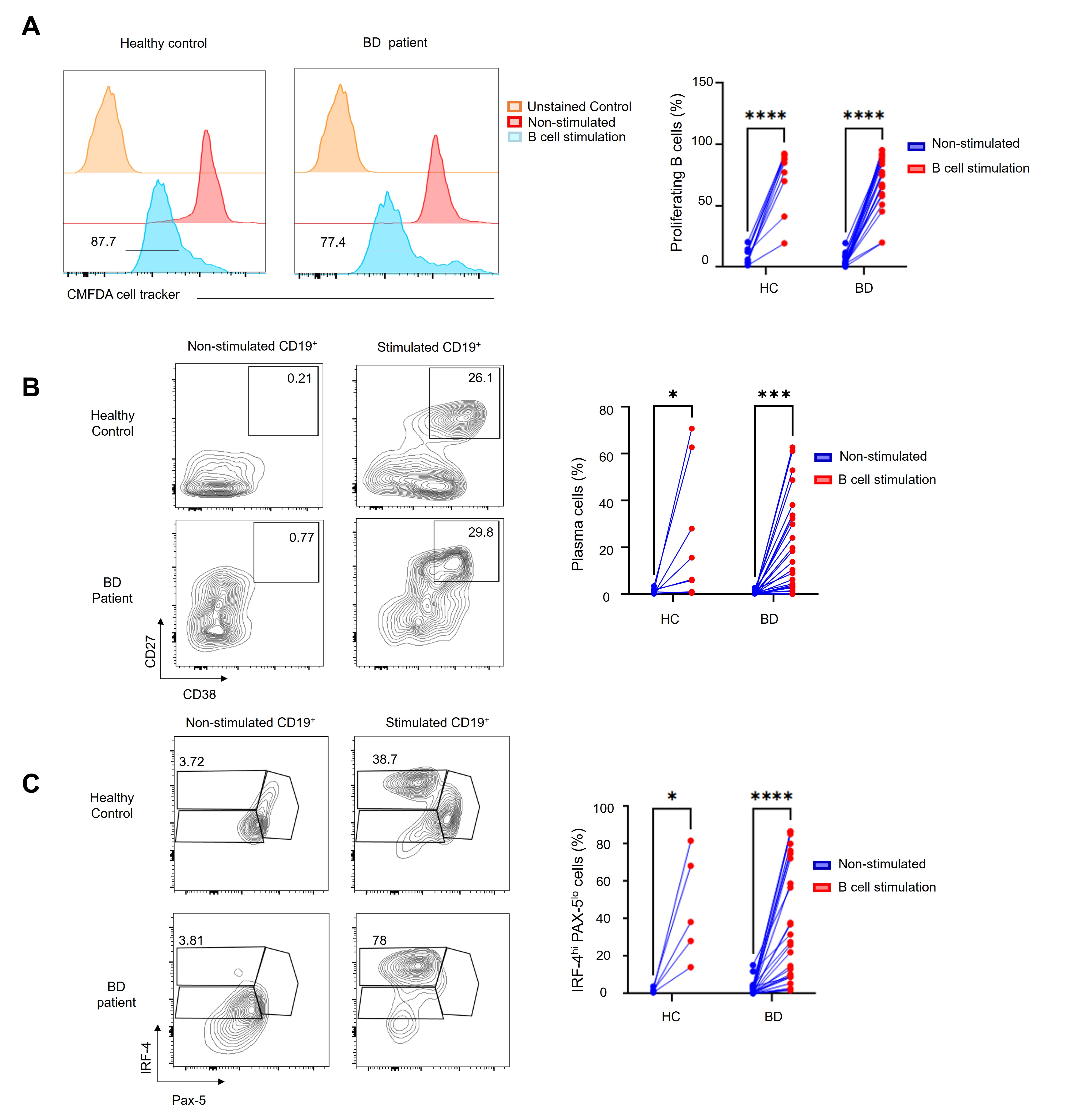

Supplement: Supplementary Figure 2 — Positive control B cell simulation during in vitro culture (A) B cell proliferation after direct stimulation in HC donors and BD patients compared to non-stimulated cells is shown in representative histograms and as individual values in symbol and line plots. Frequency of induced plasma cell (CD19+CD27+CD38hi) and IRF-4hiPAX-5lo CD19+B cells in non-stimulated versus direct B cell stimulation is shown in representative flow cytometry plots and in symbol and line plots ((B, C) respectively). Numbers on the plots and histograms represent the percentages of cell populations. *=P < 0.05; **=P < 0.01; ***=P < 0.001, ****=P < 0.0001 by 2-way ANOVA and multiple comparisons. [file Image2.jpeg]

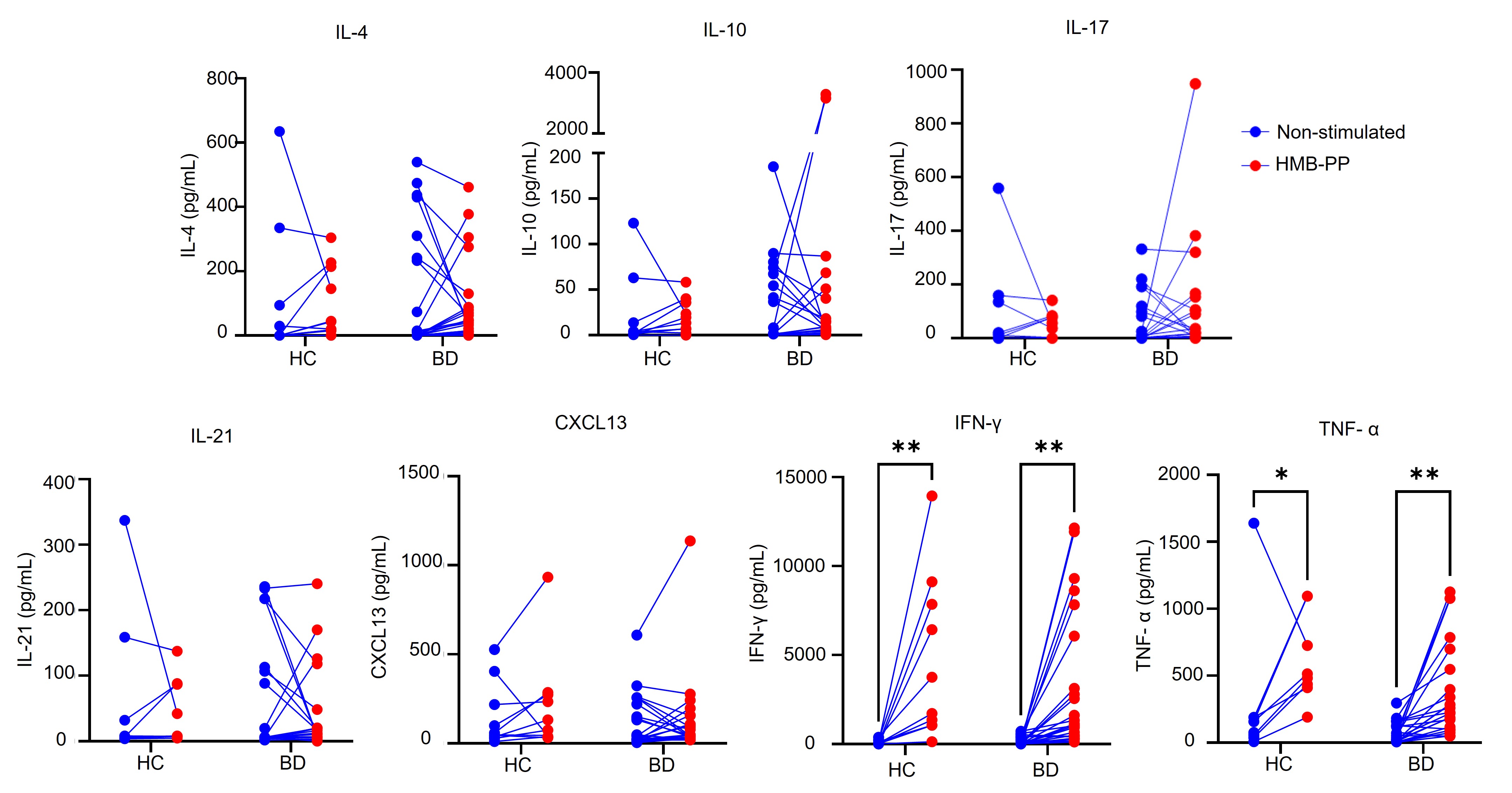

Supplement: Supplementary Figure 3 — Changes in cytokine levels after prolonged Vδ2T-cell activation Symbol and line graphs showing cytokine levels in cell culture supernatant measured after 5 days of HMB-PP stimulation in HC donors (n=11) and BD patients (n=25) relative to unstimulated control cultures. Figures show individual values. *=P < 0.05; **=P < 0.01; by 2-way ANOVA with multiple comparisons. [file Image3.jpeg]
