# Supplementary material for: Follicular helper-like γδ T cells promote plasma cell differentiation in Behçet’s disease
Source: Front Immunol. 2026 Feb 9;17:1763174. doi: 10.3389/fimmu.2026.1763174 (PMC12926121; doi:10.3389/fimmu.2026.1763174)
Supplement: Supplementary Table 1 — Flow-cytometry panel for analysis of B-cell and γδ T-cell subpopulations. [file Supplementaryfile1.docx]

**Supplementary Table 1**

**Flow-cytometry panel for analysis of B-cell and γδ T-cell subpopulations.**

| Marker | Fluorochrome | Clone | Concentration | Dilution | Company |
| --- | --- | --- | --- | --- | --- |
| CD3 | PerCP-Cy5.5 | UCHT1 | 100 μg/mL | 1:150 | BioLegend |
| CD11a | APC-Fire 750 | TS2/4 | 50 μg/mL | 1:100 | BioLegend |
| CD16 | AF700 | B73.1 | 400 μg/mL | 1:150 | BioLegend |
| CD19 | BV605 | HIB19 | 80 μg/mL | 1:100 | BioLegend |
| CD27 | BV421 | M-T271 | 100 μg/mL | 1:100 | BioLegend |
| CD28 | PE-Cy7 | CD28.2 | 150 μg/mL | 1:200 | BioLegend |
| CD56 | PE-Dazzle 594 | 5.1H11 | 100 μg/mL | 1:100 | BioLegend |
| Vδ1 | FITC | TS8.2 | 150 μg/mL | 1:100 | ThermoFisher |
| Vδ2 | PE | B6 | 100 μg/mL | 1:100 | BioLegend |
| IgD | APC | I A6-2 | 100 μg/mL | 1:150 | BioLegend |
| Live/Dead | Zombie Yellow | na | Na | 1:500 | BioLegend |

**Supplementary Table 2.**

**Flow-cytometry panel for analysis of B-cell and γδTfh-like profiles.**

| Marker | Fluorochrome | Clone | Concentration | Dilution | Company |
| --- | --- | --- | --- | --- | --- |
| CD3 | PerCP-Cy5.5 | UCHT1 | 100 μg/mL | 1:200 | BioLegend |
| CD11a | APC-Fire 750 | TS2/4 | 50 μg/mL | 1:200 | BioLegend |
| CD16 | BV650 | 3G8 | 100 μg/mL | 1:150 | BioLegend |
| CD19 | BV605 | HIB19 | 50 μg/mL | 1:150 | BioLegend |
| CD27 | BV421 | M-T271 | 100 μg/mL | 1:200 | BioLegend |
| CD28 | PE-Cy7 | CD28.2 | 150 μg/mL | 1:200 | BioLegend |
| CD56 | PE-Dazzle 594 | 5.1H11 | 100 μg/mL | 1:200 | BioLegend |
| Vδ1 | FITC | TS8.2 | 150 μg/mL | 1:100 | ThermoFisher |
| Vδ2 | PE | B6 | 100mg/mL | 1:100 | BioLegend |
| CD40L (CD154) | BV785 | 24-31 | 150 μg/mL | 1:150 | BioLegend |
| CXCR5 (CD185) | BV750 | J252D4 | 100 μg/mL | 1:150 | BioLegend |
| ICOS (CD278) | BV711 | C398.4A | 100 μg/mL | 1:150 | BioLegend |
| PD-1 | BV510 | EH12.2H7 | 150 μg/mL | 1:150 | BioLegend |
| Live/Dead | Zombie Yellow | na | Na | 1:500 | BioLegend |

**Supplementary Table 3**

**Flow-cytometry panel for analysis of cultured B-cells and γδ cells**

| Marker | Fluorochrome | Clone | Concentration | Dilution | Company |
| --- | --- | --- | --- | --- | --- |
| CD3 | PerCP-Cy5.5 | UCHT1 | 100 μg/mL | 1:100 | BioLegend |
| Vδ1 | APC | TS8.2 | 150 μg/mL | 1:50 | ThermoFisher |
| Vδ2 | PE | B6 | 100 μg/mL | 1:50 | BioLegend |
| CD40L | BV605 | 24-31 | 150 μg/mL | 1:50 | BioLegend |
| CXCR5 | BV711 | J252D4 | 100 μg/mL | 1:100 | BioLegend |
| ICOS | AF700 | C398.4A | 500 μg/mL | 1:50 | BioLegend |
| PD-1 | BV785 | EH12.2H7 | 100 μg/mL | 1:50 | BioLegend |
| Live Dead | Zombie UV | na | na | 1:500 | ThermoFisher |
| CMFDA | FITC | na | 10 mM/mL | 3 μg/mL | ThermoFisher |

**Supplementary Table 4.**

**Flow cytometry panel for analysis of plasma cells and transcription factor expression.**

| Marker | Fluorochrome | Clone | Concentration | Dilution | Company |
| --- | --- | --- | --- | --- | --- |
| CD19 | PE-Dazzle 594 | HIB19 | 50 μg/mL | 1:100 | BioLegend |
| CD27 | BV421 | O323 | 50 μg/mL | 1:100 | BioLegend |
| CD38 | PE-Cy7 | S17015A | 50 μg/mL | 1:100 | BioLegend |
| IRF-4 | PE | IRF4.3E4 | 500 μg/mL | 1:100 | BioLegend |
| Pax-5 | PerCP-Cy5.5 | 1H9 | 200 μg/mL | 1:100 | BioLegend |
| Live Dead | Zombie UV | na | na | 1:500 | BioLegend |
| CMFDA | FITC | na | 10 mM/mL | 3 μg/mL | ThermoFisher |

**Supplementary Table 5**

**Flow-cytometry panel for analysis of intracellular cytokines in γδ+T cells**

| Marker | Fluorochrome | Clone | Concentration | Dilution | Company |
| --- | --- | --- | --- | --- | --- |
| CD3 | PerCP-Cy5.5 | UCHT1 | 100 μg/mL | 1:100 | BioLegend |
| Vδ1 | APC | TS8.2 | 150 μg/mL | 1:50 | ThermoFisher |
| Vδ2 | BV510 | B6 | 100 μg/mL | 1:50 | BioLegend |
| IL-4 | PE-Cy7 | MP4-25D2 | 40 μg/mL | 1:50 | BioLegend |
| IL-21 | PE | 3A3-N2 | 50 μg/mL | 1:50 | BioLegend |
| IFNΥ | APC-Cy7 | 4S.B3 | 20 μg/mL | 1:100 | BioLegend |
| Live Dead | Zombie UV | na | na | 1:500 | ThermoFisher |

**Supplementary Table 6**

**Flow-cytometry panel for analysis of intracellular cytokines in γδ+T cells**

| Marker | Fluorochrome | Clone | Concentration | Dilution | Company |
| --- | --- | --- | --- | --- | --- |
| CD3 | PerCP-Cy5.5 | UCHT1 | 100 μg/mL | 1:100 | BioLegend |
| Vδ1 | APC | TS8.2 | 150 μg/mL | 1:50 | ThermoFisher |
| Vδ2 | BV510 | B6 | 100 μg/mL | 1:50 | BioLegend |
| IL-10 | PE-Cy7 | JES3-9D7 | 20 μg/mL | 1:50 | BioLegend |
| IL-17 | PE | eBio64CAP17 | 50 μg/mL | 1:50 | ThermoFisher |
| TNFα | APC-Cy7 | MAb11 | 20 μg/mL | 1:100 | BioLegend |
| Live Dead | Zombie UV | na | na | 1:500 | ThermoFisher |

**Supplementary Table 7.**

**Multiplex Cytokine array results (Mean ± SEM) and fold change of increase after HMB-PP stimulation.**

|  | Healthy Control | | | Behçet’s Patients | | |
| --- | --- | --- | --- | --- | --- | --- |
|  | **Non-Stimulated**  Mean ± SEM | **HMB-PP**  Mean ± SEM | **Fold Change**  **Mean** | **Non-Stimulated**  Mean ± SEM | **HMB-PP**  Mean ± SEM | **Fold Change**  **Mean** |
| TNF-α | 99.68 ± 23.88 | 816.2 ± 249.7 | **11.64** | 70.44 ± 16.24 | 365 ± 72.64 | **17** |
| IFN-γ | 90.91 ± 39.62 | 4643 ± 1439 | **118** | 164.3 ± 48.09 | 3102 ± 798.7 | **128** |
| IL-4 | 99.47 ± 61.54 | 95.56 ± 32.52 | **6.4** | 148.3 ± 48.61 | 92.21 ± 24.93 | **3.82** |
| IL-10 | 24.36 ± 11.91 | 305.5 ± 284.8 | **10.96** | 28.92 ± 9 | 273.3 ± 178 | **32.6** |
| IL-17 | 90.97 ± 50.1 | 230.3 ± 182.8 | **70.29** | 112.6 ± 43.21 | 97.89 ± 40.67 | **138.6** |
| IL-21 | 57.09 ± 34.57 | 39.71 ± 15.1 | **1.46** | 76.97 ± 26.37 | 37.85 ± 12.6 | **1.71** |
| CXCL13 | 287.1 ± 148.9 | 293.6 ± 115.4 | **1.09** | 131.7 ± 34.19 | 129.8 ± 46.16 | **1.85** |
